# Prenatal Planning and Breastfeeding: Buffering Postpartum Depression Through Positive Affect

**DOI:** 10.3390/brainsci15060591

**Published:** 2025-05-29

**Authors:** Ana Catala, Cecilia Peñacoba, Patricia Catalá

**Affiliations:** 1 Universidad Francisco de Vitoria, 28223 Pozuelo de Alarcón, Spain; anacatalame@gmail.com; 2 Department of Psychology, Universidad Rey Juan Carlos, 28922 Alcorcón, Spain; cecilia.penacoba@urjc.es

**Keywords:** prenatal planning, breastfeeding duration, postpartum depression, positive affect, maternal mental health

## Abstract

**Background/Objectives:** In the context of maternal mental health, this cross-sectional study investigates a moderated mediation model to explore how prenatal planning is associated with postpartum depression. Specifically, we examined whether planned pregnancy (X) is associated with fewer postpartum depression symptoms (Y) through greater positive affect (M), and whether the indirect association is moderated by breastfeeding duration (W). **Methods**: Data were collected from 117 postpartum mothers via self-report questionnaires that measured the degree of pregnancy planning, positive affect, postpartum depression symptoms, and breastfeeding duration. Bootstrap analyses were performed to assess the conditional indirect effects across two levels of breastfeeding duration. **Results**: The findings suggest an indirect association between pregnancy planning and postpartum depressive symptoms through positive affect, moderated by breastfeeding duration. This association was statistically significant only among mothers who breastfed for less than six months, indicating that the protective emotional effect of pregnancy planning may be more evident in this group. No significant indirect effects were observed in mothers who breastfed beyond this duration. **Conclusions**: These exploratory findings suggest that positive affect may be a pathway through which prenatal planning relates to maternal well-being, particularly in the context of breastfeeding practices. Given the cross-sectional design, causal inferences cannot be drawn. Future longitudinal research is needed to confirm these associations.

## 1. Introduction

Maternal mental health during the postpartum period is essential for the well-being of both mother and newborn, as it influences the quality of the emotional relationship and family dynamics [1]. Postpartum depression affects a significant proportion of women, impacting not only their quality of life but also their emotional bond with their baby and family dynamic [2,3]. In this context, it has been observed that mothers who experience a planned pregnancy tend to display greater emotional stability and better adaptation to motherhood [4]. This preparation, encompassing both psychological and practical aspects, can may buffer the stress and anxiety arising from the physical and hormonal changes inherent to pregnancy and the postpartum period [5,6,7].

At the same time, the duration of breastfeeding has been the subject of growing interest [8,9,10]. Various studies indicate that breastfeeding can act as a process that not only promotes the immunity and development of the newborn but also contributes to the mother’s emotional balance [11,12]. In particular, it has been observed that breastfeeding can enhance positive emotional states, characterized by optimism, energy, and personal satisfaction, essential elements for resilience to postpartum stress [13]. However, the relationship between breastfeeding duration and its effects on maternal mental health is not always linear or permanent [14].

Therefore, the objective of this study is to identify how these factors—pregnancy planning, and breastfeeding—affect maternal emotional well-being through positive affect, offering keys to the design of interventions that strengthen maternal mental health both prenatally and postpartum. In this way, we hope to contribute to an understanding of the most effective strategies for preventing or mitigating postpartum depression and, ultimately, improving the quality of life of women and their children.

## 2. Materials and Methods

### 2.1. Design and Procedure

This study adopted a cross-sectional design, evaluating 117 postpartum women. Data collection was conducted through a self-administered online questionnaire using the Microsoft Forms platform. The questionnaire was designed so that participants could not continue without completing all required fields, thus ensuring data integrity for all psychometric scales used. The questionnaire completion time was estimated to be approximately 20 min. Recruitment took place between January and February 2025 through primary care centers and digital outreach (social media, parenting forums, and institutional mailing lists).

The inclusion criteria were being over 18 years of age, having given birth between 11 and 12 months previously, and currently residing in Spain. Exclusion criteria included a self-reported diagnosis of a serious mental disorder or the inability to complete the questionnaire due to language barriers. Before beginning the questionnaire, participants were informed about the study objectives and their informed consent was obtained electronically, ensuring the voluntary nature and confidentiality of their responses. Furthermore, this study was approved by the Ethics Committee of the Rey Juan Carlos University (identification code 0103202312023), complying with all the ethical guidelines of the Declaration of Helsinki.

### 2.2. Instruments

#### 2.2.1. Planned Pregnancy

To assess whether the pregnancy was planned, a direct, ad hoc question was included. Women were asked to respond to the following: Was your pregnancy planned? Response options were “Yes” or “No”.

#### 2.2.2. Positive Affect

Positive affect was assessed using the Positive Affect Schedule (PANAS) Positive Affect scale [15]. This scale consists of 10 items in which participants indicate the degree to which they have experienced certain emotional states during the last month, using a 5-point Likert-type scale (1 = not at all or very little; 5 = very much). The total score is obtained by summing the responses to the items, generating a range of 10 to 50 points, where higher scores reflect a greater presence of positive affect. In this study, only the positive affect subscale was used. The internal consistency of the scale was high, evidenced by a Cronbach’s alpha coefficient of 0.87.

#### 2.2.3. Breastfeeding Duration

Breastfeeding duration was assessed using a direct, specific question, asking the participants to indicate the total number of months during which they breastfed their children (e.g., How many months did you breastfeed your child?). The duration of breastfeeding was self-reported by the participants. To avoid confusion regarding the use of bottles or formulas, a clarification was included in the questionnaire clarified that participants should report exclusive breastfeeding for the first six months of the baby’s life, at which point complementary foods were introduced. It was emphasized that breastfeeding continued to be the mainstay of feeding during this period. This clarification was critical to ensure that the data accurately reflected exclusive breastfeeding.

For the purpose of this study, breastfeeding duration was recoded into a dichotomous variable: ≤6 months and >6 months. This cut-off point was selected in line with recommendations by the World Health Organization [16], which recommends exclusive breastfeeding during the first six months of life, followed by continued breastfeeding alongside complementary feeding. Additionally, this threshold has been widely used in prior research as a clinically meaningful division to analyze breastfeeding-related outcomes in maternal mental health [17,18]. Categorizing the variable allowed for a more interpretable analysis of the moderating effect of breastfeeding duration in relation to maternal emotional well-being.

#### 2.2.4. Postpartum Depression

Postpartum depression was assessed using the Edinburgh Postnatal Depression Scale (EDPS) [19]. This self-report instrument consists of 10 items, in which participants indicate the frequency with which they have experienced depressive symptoms during the postpartum period, using a 4-point Likert-type scale (0 = not at all; 3 = very frequently). The sum of the item scores yields a total score ranging from 0 to 30, with higher scores reflecting greater presence of depressive symptoms. In this study, the EPDS was used to identify and quantify the severity of postpartum depression among participating mothers. The internal consistency of the scale in our sample was satisfactory, as evidenced by a Cronbach’s alpha coefficient of 0.85.

#### 2.2.5. Postpartum Pain

Postpartum pain was assessed using a numerical rating scale (NRS), asking: “How would you describe the level of physical pain you experienced during the first few weeks after delivery?” Responses ranged from 0 (“no pain”) to 10 (“the worst pain imaginable”). The NRS has shown good validity and reliability for pain assessment in clinical and perinatal populations [20].

#### 2.2.6. Sociodemographic and Occupational Variables

A specific questionnaire developed by the research team was used to collect these data. Specifically, it included age, educational level, family status (married, single, separated/divorced), employment status (teleworking, in-person work, or inactive), whether this was their first child, previous miscarriages, gestational age, and type of delivery (vaginal or cesarean).

### 2.3. Data Analysis

SPSS version 22 (IBM, Armonk, NY, USA) was used for data analysis [21]. Prior to conducting the main analyses, the assumptions of normality and homogeneity of variances were tested. The Shapiro–Wilk tests indicated that the continuous variables (positive affect, breastfeeding duration, and postpartum depression symptoms) did not follow a normal distribution (*p* < 0.05). However, Levene’s test for equality of variances yielded non-significant results (*p* > 0.05), indicating that the assumption of homoscedasticity was met.

Despite the non-normal distribution, parametric tests were deemed appropriate for the following reasons. First, our sample size (*n* = 117) meets the criteria for robustness of parametric analyses to violations of normality, especially when variances are homogeneous [22,23]. Second, the variables in this study are of a psychological nature, and it is well documented that psychological constructs (e.g., affect, mood, depression symptoms) often deviate from normality due to their intrinsic variability and measurement constraints [24,25]. In such contexts, parametric models can still produce valid and interpretable results when applied with caution.

**Figure 1:**
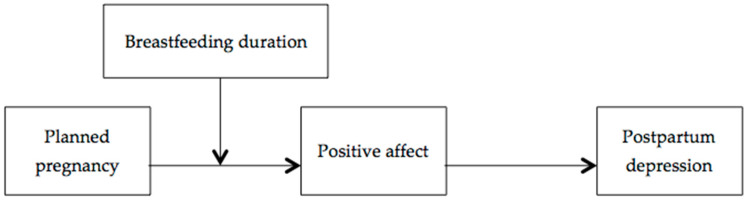
Moderated mediation model. Effect of planned pregnancy on postpartum depression through positive affect, moderated by breastfeeding duration.

First, the sample characteristics were described, and bivariate associations between the variables of interest (planned pregnancy, positive affect, breastfeeding duration, and postpartum depression) were explored. Pearson correlations were calculated for continuous variables, while Student’s *t*-test was applied for the dichotomous variable (planned pregnancy). Next, a moderated mediation model (model 7) was run using the PROCESS macro [26] to determine whether breastfeeding duration (W) moderates the indirect effect of pregnancy planning (X) on postpartum depression (Y), mediated by positive affect (M) (see Figure 1). Statistical significance was set at *p* < 0.01 (two-tailed). To assess the significance of the effects, the bootstrap method was used with 5000 samples, obtaining 95% confidence intervals. The use of PROCESS macro for SPSS [27] is compatible with non-normally distributed data. The estimation of indirect and interaction effects in PROCESS relies on bootstrapping procedures, which do not require the normality of the variables or residuals. Bootstrapping is a robust nonparametric method that provides valid confidence intervals for mediation and moderation effects, even under conditions of skewed data [28].

To control for potential confounding variables that could influence the relationship between breastfeeding duration and postpartum depression symptoms, the following covariates were included in the model: maternal age, employment status, first pregnancy and postpartum pain. These covariates were selected because previous research has shown that they can impact both breastfeeding duration and postpartum emotional well-being [29,30,31,32,33,34]. Furthermore, being a first-time mother can influence the emotional experience and how the mother handles breastfeeding and postpartum depression. Including these covariates in the model allowed for a more rigorous analysis of the relationships between the main variables, while controlling for potentially confounding effects that could alter the study’s conclusions.

## 3. Results

### 3.1. Demographic and Obstetric Profile of the Sample

The mean age of the participants was 33.04 years (SD = 4.14). Regarding marital status, 81.19% (*n* = 95) were married or in a stable relationship, 12.82% (*n* = 15) were separated or divorced, and 5.98% (*n* = 7) were single. Regarding educational level, 32.48% (*n* = 38) had university studies, 17.95% (*n* = 21) had completed a baccalaureate or vocational training, 47.01% (*n* = 55) had completed secondary education, and 2.56% (*n* = 3) had primary education. Regarding employment status, 43.59% (*n* = 51) were teleworking, 26.50% (*n* = 31) were working in person, and 29.91% (*n* = 35) were inactive.

In addition, obstetric data were collected: 24.78% (*n* = 29) of the women reported having experienced previous miscarriages. The average gestational age at delivery was 39.20 weeks (SD = 1.40). Regarding the type of delivery, 76.92% (*n* = 90) of the participants had a vaginal delivery, while 23.08% (*n* = 27) underwent a cesarean section. More than half of the participants (65.81%; *n* = 77) were experiencing motherhood for the first time. Table 1 shows a summary of the data described. The mean postpartum pain score was 5.02 (SD = 2.81).

### 3.2. Descriptive Analysis and Correlations

Table 2 summarizes the descriptive statistics and correlations of the study variables. The mean score for positive affect was 27.81 (SD = 5.78), with observed values ranging from 10 to 40. This variable was negatively associated with postpartum depression (r = −0.202, *p* < 0.05) but did not show a significant correlation with breastfeeding duration (r = 0.044). The mean duration of breastfeeding was 9.36 months (SD = 3.86), spanning from 0 to 12 months. Considering the cut-off point of six months, 30.77% (*n* = 46) of women breastfed less than six months. The duration of breastfeeding showed a moderate negative correlation with postpartum depression (r = −0.256, *p* < 0.01). Meanwhile, postpartum depression itself had a mean score of 9.43 (SD = 5.48), ranging from 3 to 50. Regarding pregnancy planning, 76.09% (*n* = 89) of participants indicated that their pregnancy was planned. Mothers whose pregnancies were planned reported significantly higher levels of positive affect (M = 28.18, SD = 5.78) compared to those with unplanned pregnancies (M = 24.58, SD = 4.94; t = −2.068, *p* = 0.041). No significant differences were found between groups regarding postpartum depression or breastfeeding duration.

### 3.3. Moderate Mediation Model

Moderated mediation analysis considered positive affect as a mediator when breastfeeding duration was used as a moderator in the relationship between planned pregnancy and postpartum depression (see Table 3). The first model, in which positive affect was considered as the dependent variable, explained 40% of the variance (R^2^ = 0.40; F = 2.38, *p* = 0.027). In this model, pregnancy planning was found to have a significant effect on positive affect (B = 11.57, t = 2.04, *p* = 0.043), as did breastfeeding duration (B = 6.29, t = 2.15, *p* = 0.033) and their interaction (B = −6.15, t = −2.01, *p* = 0.046). The included covariates (age, first-child status and employment status) did not show significant effects (*p* > 0.05) except for postpartum pain (*p* = 0.013).

In the second model, which evaluated postpartum depression as the dependent variable, it was found to explain 42% of the variance (R^2^ = 0.42; F = 3.38, *p* = 0.004). In this case, positive affect was inversely and significantly related to postpartum depression (B = −0.25, t = −2.74, *p* = 0.007), while pregnancy planning and the covariates did not show significant effects (*p* > 0.05).

**Figure 2:**
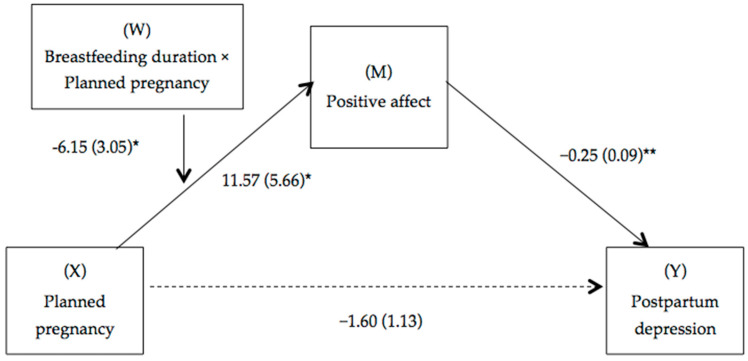
Path diagram illustrating the moderate mediation model. Notes: Values are non-standardized regression coefficients (SE in parentheses) and associated *p* values (** *p* < 0.01, * *p* < 0.05). Association in brackets = direct effect (controlling for indirect effects). Solid lines indicate important pathways, and dashed lines indicate non-significant pathways.

The moderated mediation model (see Figure 2) shows how breastfeeding duration moderates the relationship between planned pregnancy and positive affect, while positive affect mediates the relationship between planned pregnancy and postpartum depression. Specifically, mothers who planned their pregnancy experienced higher levels of positive affect, which was associated with reduced symptoms of postpartum depression. However, this beneficial effect of positive affect varied according to breastfeeding duration (Index = 1.542, SE = 0.795, 95% [CI = 0.282, 3.298]).

Table 4 shows the indirect effects of pregnancy planning on postpartum depression through positive affect, at different levels of the moderating variable (breastfeeding duration). Among mothers who breastfed for less than 6 months, the effect was statistically significant (B = −1.36, SE = 0.69, 95% CI [−2.86, −0.25]), indicating a stronger mediation effect in this subgroup. In contrast, for mothers who breastfed for more than 6 months, the indirect effect was not significant (B = 0.18, SE = 0.33, 95% CI [−0.49, 0.88]), suggesting that the mediating role of positive affect diminishes as breastfeeding extends beyond six months.

## 4. Discussion

This cross-sectional study explored a moderated mediation model to examine how pregnancy planning, positive affect, and breastfeeding duration relate to postpartum depressive symptoms. The results offer preliminary evidence suggesting that pregnancy planning may be indirectly associated with fewer depressive symptoms through higher levels of positive affect, particularly among mothers who breastfed for less than six months. In this model, positive affect may function as a potential emotional buffer, helping women navigate the demands of the postpartum period more adaptively, in line with existing literature on emotional regulation and maternal well-being [35,36,37].

Importantly, the conditional indirect effect was only statistically significant for mothers who breastfed for less than six months. This finding suggests that shorter breastfeeding durations may enhance the emotional benefits of pregnancy planning. It is possible that breastfeeding in the early months, when both emotional bonding and physiological changes are particularly intense, enhances the protective role of positive affect in reducing psychological distress. These results are consistent with studies showing that the early postpartum period is especially sensitive to the influence of psychosocial factors on maternal mental health [38,39].

Furthermore, our analysis revealed that postpartum pain was significantly associated with lower levels of positive affect. This finding highlights the potential emotional impact that physical discomfort can have on mothers during the early postpartum period. This physical discomfort possibly diminishes the capacity to experience positive emotions that protect against depressive symptoms. Although the effect of postpartum pain on postpartum depression did not reach statistical significance in the final model, the observed trend suggests a clinically relevant association that warrants further research. These results underscore the importance of addressing physical recovery and pain management in postpartum care as integral components of emotional well-being [40].

One possible explanation for the decrease in the indirect effect beyond six months lies in the timing of key transitions. Around the six-month mark, most infants begin complementary feeding [41], and many mothers return to work [42]. These changes may disrupt the dynamics of maternal caregiving and reduce the emotional reinforcement derived from breastfeeding, potentially weakening its moderating effect. In Spain, current maternity leave policies offer 16 weeks of paid leave and 28 additional days of breastfeeding leave, which often do not extend beyond this transitional period. As such, many mothers may face increased external pressures that limit their capacity to maintain breastfeeding or benefit emotionally from it during this later stage [43].

Importantly, these results should not be interpreted as suggesting that longer breastfeeding is detrimental. Rather, they highlight that the psychological impact of breastfeeding may change over time and be influenced by contextual and structural factors. From a maternal resilience perspective, fostering supportive environments and realistic expectations about caregiving roles—particularly around the six-month transition—could enhance emotional well-being [44,45].

### 4.1. Practical Implications

The findings of this study underscore the importance of integrating emotional well-being into maternal care strategies, particularly during the early postpartum period. While preliminary, these results point to the potential value of supporting pregnancy planning and fostering positive affect as protective elements for maternal mental health—especially in cases where breastfeeding lasts fewer than six months.

In this context, public health interventions could incorporate psychoeducational resources that enhance emotional regulation, self-efficacy, and meaning-making in the postpartum phase. Additionally, providing mothers with realistic guidance on breastfeeding—acknowledging the variability of each case—may help reduce the pressure to conform to rigid expectations and instead promote flexible, informed decision-making aligned with each woman’s context.

Creating supportive work environments and family policies that accommodate diverse breastfeeding trajectories—regardless of duration—can also play a role in maintaining maternal well-being. Information campaigns might focus not only on the benefits of breastfeeding for the infant but also on how the early months of feeding and bonding may intersect with emotional experiences in the mother. Highlighting the psychological dimension of early caregiving could contribute to a more holistic and compassionate approach to postpartum support.

In addition, the observed association between postpartum pain and reduced positive affect emphasizes the need to address physical recovery as a critical element of emotional support. Postpartum care protocols should not only focus on infant-related outcomes but also include strategies for managing maternal discomfort and pain, which could play a significant role in promoting emotional resilience and preventing affective symptoms during this vulnerable period.

### 4.2. Limitations

A key limitation of this study is its reliance on cross-sectional data, which precludes any definitive conclusions regarding causality. While valuable insights were gained, the inability to establish the direction of causality remains an issue. Mediation analyses conducted in cross-sectional studies may lead to problems of temporal ambiguity. Furthermore, as noted in previous studies [46], mediation in cross-sectional data may underestimate or distort indirect effects that could be observed with a longitudinal design, thus affecting the interpretation of the strength of causal relationships. Because the model presented in this study does not allow determining the temporal order of events, the use of longitudinal data in future research would be essential to validate the causal relationships suggested in this analysis. Furthermore, the percentage of women who were actually able to breastfeed until six months may be limited by socio-occupational or personal circumstances. The use of self-reports, meanwhile, may lead to social desirability bias. Future research could use longitudinal methodologies to delve deeper into the mothers’ emotional journeys and unravel in more detail the dynamics between pregnancy planning, breastfeeding, and positive affect. Furthermore, the mediation model presented in this study does not consider measurement error in key variables, such as positive affect and postpartum depression, which could bias indirect effects and the estimation of moderation effects. Measurement error is particularly relevant in studies using self-report scales, such as the PANAS and EPDS, as participants may misinterpret questions or exhibit response bias. Future studies would benefit from implementing more robust measurement methods, such as multidimensional assessment or the use of latent variables, to reduce the impact of measurement error and improve the reliability of the results. This would also help mitigate biases that can occur when moderated mediation models are used without accounting for measurement error in the variables involved.

### 4.3. Future Research

Future research should employ longitudinal designs to further explore the dynamic interplay between pregnancy planning, breastfeeding duration, and maternal mental health. Given that the protective indirect effect identified in this study was limited to breastfeeding durations of less than six months, it would be important to examine why this window appears to be particularly significant for maternal emotional well-being. Investigating whether breastfeeding beyond this period maintains, reduces, or modifies these associations could offer valuable insight into the evolving emotional landscape of motherhood.

Additionally, expanding the scope to include a broader range of socioeconomic, occupational, and contextual variables—such as support networks, maternity leave policies, and maternal caregiving load—could deepen our understanding of how structural factors influence emotional outcomes in the postpartum period. Such research would be instrumental in informing evidence-based public health strategies aimed at promoting maternal mental health through personalized and context-sensitive interventions.

## 5. Conclusions

This study provides exploratory evidence suggesting that pregnancy planning and positive affect may be protective factors associated with lower postpartum depressive symptoms, particularly among women who breastfed for less than six months. The observed indirect association highlights a potentially sensitive period during which the emotional benefits of prenatal planning may be amplified by early breastfeeding experiences.

These findings should be interpreted as preliminary and descriptive, as the cross-sectional design does not allow for causal inferences. Rather, the present work serves as a proof-of-concept study that illustrates possible relational pathways between psychological and behavioral factors in the postpartum period. Future longitudinal research is needed to clarify the temporal sequencing and underlying mechanisms that link pregnancy planning, emotional well-being, breastfeeding duration, and postpartum depression.

Finally, the trend observed between postpartum pain and depressive symptoms, along with its significant effect on positive affect, suggests that maternal physical discomfort may be a relevant yet underrecognized factor in postpartum emotional well-being. These findings advocate for an integrated approach to maternal health that considers both emotional and physical dimensions in postpartum care.

## Figures and Tables

**Table 1:** Description of the sample.

Variable	Frequency (*n*) or Mean (SD)	Percentage (%)
Mean age	33.04 years (SD = 4.14)	
Marital status		
Married or in a stable relationship	95	81.19%
Separated or divorced	15	12.82%
Single	7	5.98%
Educational level		
University studies	38	32.48%
Baccalaureate or vocational training	21	17.95%
Completed secondary education	55	47.01%
Primary education	3	2.56%
Employment status		
Teleworking	51	43.59%
In-person work	31	26.50%
Inactive	35	29.91%
Previous miscarriages	29	24.78%
Mean gestational age at delivery	39.2 weeks (SD = 1.40)	
Type of delivery		
Vaginal delivery	90	76.92%
Cesarean section	27	23.08%
First-time motherhood	77	65.81%
Postpartum pain	5.02 (SD = 2.81)	

**Table 2:** Descriptives of psychosocial characteristics.

Psychosocial Characteristics	Mean (SD)	Sample Range	Correlations	
			Breastfeeding Duration	Postpartum Depression
Positive affect	27.81 (5.78)	10–40	0.044	−0.202 *
Breastfeeding duration	9.36 (3.86)	0–12		−0.256 **
Postpartum depression	9.43 (5.48)	3–30		
Breastfeeding duration, *n* (%)				
≤6 months	46 (30.77)			
>6 months	81 (69.23)			
Planned pregnancy, *n* (%)				
Yes	89 (76.09)			
No	28 (23.93)			

Note: SD (standard deviation); *n* (number); % (percentage); ** *p* < 0.01, * *p* < 0.05

**Table 3:** Moderate mediation analysis.

VD: Positive Affect	*R* ^2^	*F*	*p*	Beta	*t*	*p*
Model summary	0.40	2.38	0.027			
VI: Planned pregnancy				11.57	2.04	0.043
W: Breastfeeding duration				6.29	2.15	0.033
Planned pregnancy × Breastfeeding duration				−6.15	−2.01	0.046
Age (covariate)				0.07	0.88	0.380
First child (covariate)				0.32	0.39	0.696
Employment status (covariate)				0.44	1.23	0.219
Postpartum pain (covariate)				−0.43	−2.51	0.014
**VD: Postpartum Depression**	***R* ^2^**	***F***	***p***	**Beta**	***t***	***p***
Model summary	0.42	3.38	0.004			
VI: Planned pregnancy				−1.60	−142	0.157
M: Positive affect				−0.25	−2.74	0.007
Age (covariate)				−0.01	0.02	0.998
First child (covariate)				−0.50	−0.64	0.523
Employment status (covariate)				−0.13	−0.41	0.675
Postpartum pain (covariate)				0.31	1.94	0.059

**Table 4:** Indirect conditional effect at specific levels of the moderator.

Breastfeeding Duration	Beta	SE	LL 95% CI	UL 95% CI
≤6 months	−1.36	0.69	−2.86	−0.25
>6 months	0.18	0.33	−0.49	0.88

Notes: SE = standard error; LL95%CI = lower level of the 95% confidence interval; UL95%CI = upper level of the 95% confidence interval.

## Data Availability

The data presented in this study are available on request from the corresponding author. The data are not publicly available due to privacy restrictions.
